# Gene Polymorphism of Toll-Like Receptors and Lung Function at Five to Seven Years of Age after Infant Bronchiolitis

**DOI:** 10.1371/journal.pone.0146526

**Published:** 2016-01-07

**Authors:** Eero Lauhkonen, Petri Koponen, Juho Vuononvirta, Johanna Teräsjärvi, Kirsi Nuolivirta, Jyri O. Toikka, Merja Helminen, Qiushui He, Matti Korppi

**Affiliations:** 1 Tampere Center for Child Health Research, Tampere University and University Hospital, Tampere, Finland; 2 Department of Medical Microbiology and Immunology, Turku University, Turku, Finland; 3 Department of Infectious Disease Surveillance and Control, National Institute for Health and Welfare, Turku, Finland; 4 Seinäjoki Central Hospital, Seinäjoki, Finland; 5 Department of Clinical Physiology, Tampere University Hospital, Tampere, Finland; 6 Department of Clinical Physiology, Turku University Hospital, Turku, Finland; IISER-TVM, INDIA

## Abstract

**Aim:**

Toll-like receptors (TLR) play a crucial role in innate immunity, protecting the host from pathogens such as viruses. Genetic variations in TLRs have been associated with the severity of viral bronchiolitis in infancy and with the later occurrence of post-bronchiolitis asthma. The aim of the present study was to evaluate if there are any exploratory associations between *TLR* gene polymorphisms and lung function at 5 to 7 years of age in former bronchiolitis patients.

**Methods:**

We performed impulse oscillometry (IOS) at the median age of 6.3 years for 103 children who had been hospitalized for bronchiolitis at less than six months of age. The main parameters evaluated were airway resistance and reactance at 5Hz in baseline and post-exercise measurements. Data on single nucleotide polymorphisms (SNP) of *TLR1* rs5743618, *TLR2* rs5743708, *TLR6* rs5743810 and *TLR10* rs4129009 (TLR2 subfamily) and *TLR3* rs3775291, *TLR4* rs4986790, *TLR7* rs179008, *TLR8* rs2407992 and *TLR 9* rs187084 were available for analyses.

**Results:**

The *TLR4* rs4986790 wild genotype A/A was associated with a greater Rrs5 response (0.72 vs. -0.42, p = 0.03) to exercise. In *TLR6* rs5743810, the minor allele T was associated with greater Rrs5 response (0.80 vs. -0.03, p = 0.04) to exercise. In *TLR7* rs179008, the major allele A was associated with baseline decline in dRrs/df (-1.03 vs 0.61, p = 0.01) and increased Fres (2.28 vs. 0.89, p = 0.01) in girls.

**Conclusion:**

Among the nine studied TLRs, only *TLR7* rs179008 showed some exploratory associations with post-bronchiolitis lung function deficiency, and polymorphisms of *TLR4* rs4986790, and *TLR6* rs5743810 in particular, with airway reactivity. These findings call for further confirmatory studies.

## Introduction

In a recent Finnish study, on average 37 per 1000 infants under the age of six months were annually admitted to the emergency room due to viral bronchiolitis and 70% of them were hospitalized. [1] At this age, respiratory syncytial virus (RSV) is the predominant cause of bronchiolitis, and most of the affected infants have no previous medical history. [2,3] Toll-like receptors (TLR), which recognize pathogens and initiate responses of both innate and adaptive immunity, are the gatekeepers of the immune system. [4] TLRs are important in airway mucosal responses to acute viral infections [5] and in later emergence and regulation of asthmatic inflammation. [6]

In the respiratory epithelial cells, TLRs 1, 2, 4, 6 and 10 are expressed on the cell surfaces. TLRs 1, 2, 6 and 10 are co-receptors that form a functional unit called the TLR2 subfamily encoded by genes in the chromosome 4, and play an important role in recognition of bacterial structures. [7] TLRs 3, 7, 8 and 9 are endosomal receptors that recognize viral RNA and DNA structures. [5] Bacterial lipopolysaccharide is the main ligand for TLR 4, but also the F-protein of the RSV is recognized by TLR 4, being highly expressed in airway epithelium during RSV infection promoting inflammation. [5,8] The genetic alterations in TLRs were associated with the severity of bronchiolitis in infancy [8] and the later development of asthma. [9] Attenuated TLR signaling and altered antigen presenting cell function can lead to more severe infection with a longer duration due to less viral clearance. [10] On the other hand, attenuated TLR signaling could lead to repeated viral infections, which may further increase the risk of later atopy. [10,11] Thus, genetic differences in the TLR function might be associated with direct lung injury during bronchiolitis, or contribute to the development of later lung function reduction.

Impulse oscillometry (IOS), [12] which measures lung function during tidal breathing, is a promising technique that can be used for preschool-aged children who are unable to perform the maximal expiratory blow needed in spirometry. [13] The method is also appropriate for the evaluation of airway reactivity to exercise and responses to bronchodilators. [14,15] Finnish age-specific height-adjusted reference values for IOS parameters were published recently. [16]

This study focused on children who had been hospitalized for bronchiolitis at less than 6 months of age. The aim of this exploratory study was to see whether we could find any associations between the single nucleotide polymorphisms (SNP) at *TLR1* rs5743618, *TLR2* rs5743708, *TLR3* rs3775291, *TLR4* rs4986790, *TLR6* rs5743810, *TLR7* rs179008, *TLR8* rs2407992, *TLR9* rs187084 or *TLR10* rs4129009 and lung function or airway reactivity measured by IOS at 5 to 7 years of age.

## Materials and Methods

### Design

As previously published, we prospectively followed-up a cohort of 166 children hospitalized for bronchiolitis at less than 6 months of age until they were 5 to 7 years of age. [17] Of these, 127 attended a clinical follow-up visit at a median age of 6.3 years, [17] and 107 children aged less than 7 years performed impulse oscillometry (IOS). Four children were excluded from the IOS analysis for technical reasons. Body mass index (BMI) was calculated and expressed as z-scores from the population means (zBMI) and one child was excluded from the analyses due to pathologically low BMI (<16 kg/m^2^) at follow up. This meant that IOS data on 102 children were available for the analysis and data on SNPs of TLR 1, 2, 3, 4, 6, 7 and 8 were available for 98 and of TLR 9 and 10 for 97 of them.

Respiratory syncytial virus (RSV) etiology of bronchiolitis was registered while the infant was hospitalized for bronchiolitis. [18] Consumption of inhaled corticosteroids (ICS) (present in 12 cases) and presence of atopic eczema (present in 31 cases) currently and during the preceding 12 months were asked and registered at the controls visit at 5 to 7 years of age.

### Impulse oscillometry

The IOS method [19] measures total airway impedance (Zrs) resulting from phase and pressure changes of the airflow when oscillation at 1–35 Hz frequency is conducted to the bronchial tree during quiet breathing. The lower (<15 Hz) frequencies vibrate the peripheral and higher frequencies the more central airways. The main clinical parameters airway resistance (Rrs) and airway reactance (Xrs) are derived from the Zrs mathematically. Rrs describes the resistive forces to the airflow and Xrs the elastic forces of the tissues surrounding the moving air column. Additional parameters describing the change in bronchial tone and recoil as a function of the oscillation frequency are the frequency dependency of resistance (dRs/df), and the resonant frequency (Fres), a point where the resistive and elastic forces equal each other. As an example, in small airway obstruction, as in asthma, resistance at 5Hz increases above normal and the frequency dependency of resistance (dRs/df) becomes more negative, and the Xrs decreases and as a result the Fres rises. [15] Rrs and Xrs can change somewhat independently, as they are vector components of the measured Zrs in a mathematical sense. [19] Thus different lung pathology can be described with these IOS parameters, as for example reactance at 5Hz decreases in both peripheral obstruction and in restrictive conditions, as in hyperinflation or fibrosis. [15]

### Lung function measurement and analysis

First, the baseline IOS (Masterscreen IOS, Jaeger, Hochberg, Germany) measurements were obtained and pre-analyzed by an experienced clinical physiologist (JOT) to ensure they were graphically appropriate, free from artefacts and coherent in set criteria (>0.6 at 5Hz and >0.9 at 10Hz) [14,15].

Then, the children performed an outdoor free running exercise challenge test (ECT) for 8 minutes at >90% of expected heart rate (205-age/2) using a heart rate monitor (Polar, Kempele, Finland) and post-exercise IOS measurements were taken in a manner identical to the baseline measurements.

Finnish population-based, height-adjusted, age-specific reference values were published recently [16] and we used these to calculate all baseline and post-exercise IOS results as height-adjusted z-scores in resistance at 5 Hz (Rrs5), reactance at 5 Hz (Xrs5), frequency dependency of resistance (dRs/df) and resonant frequency (Fres). For the main parameters Rrs5 and Xrs5 a z-score cut-off 1.65SD is considered pathological in the baseline measurement. [16] Bronchial hyper-reactivity was evaluated as changes (Δ) in z-scores of Rrs5 and Xrs5.

As previously published [20], either Rrs5 or Xrs5 was pathological in the baseline IOS in 21 study subjects (20.4%), compared to the reference values. [16] Baseline Rrs5 was pathological in 8 (7.8%) cases and Xrs5 in 19 (18.4%) cases. Bronchial reactivity was considered pathological if the post-exercise Rrs5 change was 35% or more [14,21] and such hyper-reactivity was seen in 5 (4.9%) cases. Irreversible pathological changes of resistance or reactance at 5Hz were only seen in one child.

### Genetic studies

The genotyping of SNPs *TLR1* rs5743618, *TLR2* rs5743708 and *TLR 6* rs5743810 has previously been described in detail. [22,23] Polymorphisms of *TLR3* rs3775291 (1234 C/T), *TLR4* rs4986790 (1194 A/G), *TLR7* rs179008 **(**171 A/T), *TLR8* rs2407992 (2040 C/G), *TLR9* rs187084 (1486 T/C) and *TLR10* rs4129009 (2322 A/G) were selected due to their evident functional properties. (Table 1) The genotyping of *TLR3* rs3775291 (1234 C/T) was performed by pyrosequencing (PSQ™96MA Pyrosequencer, Biotage, Uppsala, Sweden), using a PSQ™96 Pyro Gold Q96 reagent kit according to the manufacturer's protocol. [24] The genotyping of *TLR4* rs4986790 (299 A/G) was performed by pyrosequencing with the ABIPRISM 7000 Sequence Detection System (Applied Biosystems, CA), [25] supplemented later with pyrosequencing (PSQ™96MA Pyrosequencer, Biotage, Uppsala, Sweden), using a PSQ™96 Pyro Gold Q96 reagent kit. [26,27] The genotyping of *TLR8* rs2407992 (2040 C/G) was performed in the same manner as described for *TLR3* rs3775291 (1234 C/T). [24] For *TLR7* rs179008 (171 A/T), the PCR products were first purified using the QIA quick PCR Purification Kit (Qiagen, Hilden, Germany) according to the manufacturer's protocol. PCR products with low deoxyribonucleic acid (DNA) content were eluted to 30μl of elution buffer. After purification, the PCR products were pipetted to 96-well plate (5μl) together with *TLR7* rs179008 **(**171 A/T) forward primer (1.6μl) and the 96-well plate was sent to the Institute for Molecular Medicine laboratory in Helsinki, Finland, for sequencing. *TLR9* rs187084 (1486 T/C) genotyping was performed according to Etem et al [28] by using BspTI restriction enzyme (ThermoFischer Scientific, Waltham, USA) for digestion of PCR product. High resolution melting analysis (HMRA) (Roche Diagnostics Light Cycler 480, Basel, Switzerland), was used for genotyping of *TLR10* rs4129009 (2322 A/G). HMRA PCR reactions were run at 95°C for 10 min followed by 45 cycles amplification at 95°C for 10 s, at 59°C for 10 s and at 72°C for 15 s. After PCR process final melting cycle conditions were as outlined by Roche: first heating to 95°C and hold for 1 min, cooling to pre-hold temperature (40°C) to make sure that all PCR products have re-associated and encourages heteroduplex formation. Melting interval for collecting fluorescence from 60°C -95° at ramp rate 0.02°C per second. In each run, known *TLR10* rs4129009 standards (wild type, heterozygote and homozygote) were used.

**Table 1:** Primer sequences, allele and amino acid changes in toll-like receptor (TLR) genes *TLR7* rs179008 (171A/T), *TLR8* rs2407992 (2040C/G), *TLR9* rs187084 (1486T/C) and *TLR10* rs4129009 (2322A/G).*

**TLR7 11Gln>Leu (171A/T) (rs179008)**
for 5´-AGATGTCTGGTATGTGGTT-3´
rev 5´-TGATTCTTGGTATGTTTTAGA-3´
**TLR8 651Leu>Leu (2040C/G) (rs2407992)**
for 5´-TGGAAAGCAAGTCCCTGGTA-3´
rev 5´-Biotin-AGTGAGACTCGCTGGCAAAT-3´
seq 5´-ATCCCTTAATAGGCT-3´
**TLR9 (silent mutation) (1486T/C) (rs187084)**
for 5´-ACTATGGAGCCTGCCTGCCATGATACC-3´
rev 5´-ATCCAGCCTTCTTACAAACCTCCCACC-3´
restriction enzyme BspTI
**TLR10 775Ile>Val (2322A/G) (rs4129009)**
for 5´-CTTACTGGAACCCATTCCATTCTATTGC-3´
rev 5´-TCAATGTACATCCCAACAGTGTATGTGG-3´
restriction enzyme VspI
**TLR10 775Ile>Val (2322A/G) (rs4129009)**
for 5´-AGTTCATACATTTCTCTGGTGGCT-3´
rev 5´-GTGGGCTTTTCTGGGCAAAC-3´

*TLR7 SNP was detected by common sequencing, *TLR8 SNP by pyrosequencing*, *TLR9 by digestion and TLR10 by high resolution analysis*

The PCR and sequencing primers used for the *TLR7*, *TLR8*, *TLR9* and *TLR10* genes are described in Table 1. The primers were purchased from Sigma-Aldrich, Finland.

### Statistics

SPSS Statistical Package Version 21 (IBM, NY, USA) was used in the statistical analyses of the data. The IOS outcome parameters were graphically estimated to be normally distributed. The results are expressed as means, standard deviations (SD) and 95% confidence intervals (95% CI) for continuous variables and as numbers and frequencies for categorized variables. Chi-square and Fisher’s exact tests were used in the analyses of categorized data. Analysis of co-variance (ANCOVA) was used in the analyses of continuous data and, when appropriate, adjusted for age, sex, RSV vs. non-RSV etiology of bronchiolitis, current BMI z-score, current atopic eczema and the use of ICS medication during the last 12 months. The p-value <0.05 was considered statistically significant.

Since the *TLR7* rs179008 and *TLR8* rs2407992 genes are located in the X chromosome, the analyses were carried out separately for 48 boys and 50 girls. When the data from the boys and girls were analyzed together, the male allele carries were combined with female homozygotes or, alternately, with female heterozygotes.

Further genotype combination analyses were carried out for the TLR2 subfamily (*TLR1* rs5743618, *TLR2* rs5743708, *TLR6* rs5743810 and *TLR10* rs4129009).

### Ethics

The study was approved by the Ethics Committee of the Tampere University Hospital District, approval number R13025. Informed written consent was obtained from the parents before we enrolled the infants hospitalized for bronchiolitis in infancy and at the control visit at the age of 5 to 7 years. Genetic studies were carried out anonymously and were limited to the evaluation of asthma risk.

## Results

The genotypes, presence of major and minor alleles as homozygotes and herezygotes, and minor allele frequencies (MAF) of TLRs 1, 2, 3, 4, 6, 7, 8, 9 and 10 in 98 children hospitalised for bronchiolitis in infancy and MAFs in the Finnish population are presented in Table 2. The MAFs for all nine TLR SNPs in our patients were surprisingly similar to those in the non-selected Finnish population.

**Table 2:** Toll-like receptor 1, 2, 3, 4, 6, 7, 8, 9 and 10 genotype and minor allele frequencies in 98 children hospitalised for bronchiolitis and the Finnish population.

SNP (Major>Minor)	Major/Major	Major/Minor	Minor/Minor	MAF	FIN
	(Wild*)	(Variant*)	(Variant*)		
TLR1 rs5743618 (G>T)	0.78	0.18	0.04	0.13	0.17
TLR2 rs5743708 (C>T)	0.94	0.06	0	0.03	0.03
TLR3 rs3775291 (C>T)	0.49	0.41	0.10	0.31	0.33
TLR4 rs4986790 (A>G)	0.85	0.15	0	0.08	0.12
TLR6 rs5743810 (C>T)	0.31	0.45	0.24	0.47	0.42
TLR7 rs179008 (A>T)	0.62 (girls)	0.32 (girls)	0.06 (girls)	0.27 (all)	0.31
	0.81 (boys)	0	0.19 (boys)		
TLR8 rs2407992 (G>C)	0.38 (girls)	0.42 (girls)	0.20 (girls)	0.42 (all)	0.36
	0.56 (boys)	0	0.44 (boys)		
**TLR9 rs187084 (T>C)	0.30	0.44	0.26	0.48	0.45
**TLR10 rs4129009 (A>G)	0.85	0.14	0.01	0.08	0.08

*Wild genotype means that minor allele is not presented and variant genotype that minor allele presents either as heterozygous or homozygous, *MAF = minor allele frequency*, *FIN = Finnish MAFs as in* [29]

**n = 97 for TLR9 and TLR10.

### TLR 4

In *TLR4* rs4986790, the wild A/A genotype was significantly associated with a greater response to exercise in Rrs5 (0.72 *vs*. -0.42 in those with A/G genotype, p = 0.03). (Table 3) Since there were no cases with the homozygous variant G/G genotype, separate analyses based on the presence or absence of the A or T allele in the child were not possible.

**Table 3:** *TLR4* rs4986790 genotypes and baseline IOS (z-scores) and exercise-induced changes (z-scores) in the 98 former bronchiolitis patients.

Parameter	A/A (wild)	p-value*	A/G (variant)
	n = 83		n = 15
	Mean (SD)		Mean (SD)
Rrs5	-0.13 (1.07)	0.15	0.35 (0.91)
Xrs5	-0.74 (1.28)	0.82	-0.66 (0.90)
Fres	2.20 (0.79)	0.43	2.46 (1.05)
dRrs/df	-1.03 (1.15)	0.93	-1.03 (0.96)
ΔRrs5	0.72 (1.81)	**0.03**	-0.42 (1.84)
ΔXrs5	-0.39 (1.35)	0.40	-0.10 (0.90)

Rrs5 = Resistance at 5 Hz; Xrs5 = Reactance at 5 Hz; Fres = resonant frequency; dRs/df = Frequency dependency of resistance

*A/A vs. *A/G*, *adjusted for age*, *sex*, *RSV etiology of bronchiolitis*, *current BMI z-score*, *current atopic eczema and use of inhaled corticosteroids during the preceding 12 months*. *p<0*.*05 marked in bold*.

### *TLR2* subfamily

In *TLR1* rs5743618 and *TLR2* rs5743708, there were no significant associations between genotypes or presence or absence of major or minor alleles and IOS parameters at baseline or presence or absence of bronchial hyper-reactivity. (Data not shown)

In *TLR6* rs5743810, there were no significant associations between genotypes and IOS parameters at baseline or presence of hyper-reactivity. (Data not shown) However, the presence of the minor T allele was significantly associated with greater responses to exercise in Rrs5 (increase in resistance), when compared with those without T allele. In line, there was a trend that responses in Xrs5 (decrease in reactance) were greater if the minor T allele was present (Table 4)

**Table 4:** Presence of *TLR6* rs5743810 genotypes (wild vs. variant) and baseline IOS (z-scores) and exercise-induced changes (z-scores) in the 98 former bronchiolitis patients.

Parameter	C/T or T/T (variant)	p-value*	C/C (wild)
	n = 68		n = 30
	Mean (SD)		Mean (SD)
Rrs5	-0.08 (1.05)	0.62	0.00 (1.07)
Xrs5	-0.71 (1.26)	0.78	-0.77 (1.17)
Fres	2.28 (0.69)	0.55	2.15 (1.10)
dRrs/df	-1.06 (1.05)	0.95	-0.99 (1.26)
ΔRrs5	0.80 (1.90)	**0.04**	-0.03 (1.61)
ΔXrs5	-0.52 (1.37)	0.08	0.05 (0.98)

Rrs5 = Resistance at 5 Hz; Xrs5 = Reactance at 5 Hz; Fres = resonant frequency; dRrs/df = frequency dependency of resistance.

*T-allele present vs. *T-allele not present*, *adjusted for age*, *sex*, *RSV etiology of bronchiolitis*, *BMI z-score*, *current atopic eczema and use of inhaled corticosteroids during the preceding 12 months*. *p<0*.*05 marked in bold*.

The joint analyses of the *TLR1* rs5743618, *TLR2* rs5743708, *TLR6* rs5743810 and *TLR10* rs4129009 genes were carried out for 97 cases with complete data available, and the analyses were adjusted for age, sex, RSV etiology of bronchiolitis, current BMI z-score, current atopic eczema and the use of ICSs during the last 12 months. Of the 16 possible combinations of wild or variant genotypes, 8 were present in the study population. (Table 5) We found that 20 children had the combination of four wild genotypes as homozygous, and this combination was associated with a smaller response to exercise in Rrs5 (-0.17 *vs*. 0.73, p = 0.049) compared to those 77 with a combination of one or more variant genotypes. In addition, there was a trend of improving Xrs5 (0.12 vs. -0.48, p = 0.07), but the association was not statistically significant. The combination that included the wild *TLR1* rs5743618, *TLR2* rs5743708 and *TLR10* rs4129009 genotypes, together with the variant *TLR6* rs5743810 genotype, was present in 50 children and was associated with a greater response to exercise in Rrs5 (0.91 vs. 0.16, p = 0.043) compared to those 47 children with other genotype combinations. Any other combination of genotypes, including one, two, three or four variant genotypes, did not show significant associations with IOS results. (Table 5)

**Table 5:** The joint analyses of the *TLR1* rs5743618, *TLR2* rs5743708, *TLR6* rs5743810 and *TLR10* rs4129009 genotype combinations and baseline (z-scores) and hyper-reactivity IOS measurements (Δz-scores) in 97 children hospitalized for bronchiolitis.

*TLR1/TLR2/TLR6/TLR10*	n	Rrs5	Xrs5	Fres	dRrs/df	ΔRrs5	ΔXrs5
genotype combination		Mean	Mean	Mean	Mean	Mean	Mean
		SD	SD	SD	SD	SD	SD
wild/wild/wild/wild	20	0.14	-0.81	2.31	-1.22	-0.17*	0.12
		(1.19)	(1.19)	(1.05)	(1.40)	**(1.70)**	(1.16)
variant/wild/wild/wild	3	-0.21	-0.64	2.12	-0.94	0.20	-0.21
		(0.77)	(0.39)	(0.12)	(0.31)	(1.07)	(0.68)
wild/wild/variant/wild	50	-0.26	-0.71	2.23	-1.01	0.91**	-0.53
		(1.08)	(1.38)	(0.69)	(1.09)	**(1.87)**	(1.30)
variant/wild/variant/wild	3	0.86	-1.38	3.02	1.93	1.47	-1.44
		(1.07)	(0.75)	(0.93)	(0.80)	(2.83)	(3.44)
variant/wild/wild/variant	7	-0.31	-0.71	1.69	-0.35	0.25	-0.06
		(0.74)	(1.43)	(1.42)	(0.86)	(1.67)	(0.44)
wild/variant/variant/wild	5	0.64	-0.64	2.29	-0.89	-0.37	-0.17
		(0.41)	(0.91)	(0.50)	(1.18)	(0.98)	(0.87)
variant/wild/variant/variant	8	0.14	-0.52	2.28	-1.20	0.65	-0.48
		(0.93)	(0.94)	(0.72)	(0.95)	(2.42)	(1.36)
variant/variant/variant/wild	1	0.89	-0.85	2.59	-1.09	0.81	-0.78

Rrs5 = Resistance at 5 Hz; Xrs5 = Reactance at 5 Hz; Fres = Resonant frequency, dRs/df = Frequency dependency of resistance

*p = 0.*049*

**p = 0.*043 vs*. *all other genotype combinations*, *as adjusted for age*, *sex*, *RSV etiology of bronchiolitis*, *current BMI z-score*, *current atopic eczema and use of inhaled corticosteroids during the preceding 12 months*.

### *TLR 3*, *7*, *8* and *9*

In *TLR3* rs3775291, there were no significant associations between genotypes or alleles and IOS measurements of baseline lung function or bronchial hyper-reactivity. (Data not shown) In *TLR7* rs179008, the test result showed heterozygosity A/T in one boy, and the case was deleted from the analyses. There were no significant associations between the presence of A or T alleles and any IOS parameter in boys (Data not shown).

The girls with the *TLR7* rs179008 variant heterozygous A/T genotype had the highest Fres and lowest dRrs/df in baseline IOS measurements, but there were no significant findings in hyper-reactivity parameters. (Table 6)

**Table 6:** *TLR7* rs179008 genotypes and baseline IOS (z-scores) and exercise-induced changes (z-scores) in the 50 former bronchiolitis cases who were girls.

Parameter	A/A (wild)	A/T (variant)	T/T (variant)	p-value*
	n = 31	n = 16	n = 3	
	Mean (SD)	Mean (SD)	Mean (SD)	
Rrs5	-0.26 (0.96)	0.28 (1.08)	-0.12 (1.10)	0.30
Xrs5	-0.57 (1.14)	-0.90 (0.78)	0.49 (0.56)	0.17
Fres	2.15 (0.84)	2.53 (0.59)	0.89 (1.38)	**0.02**
dRrs/df	-0.85 (0.82)	-1.36 (1.34)	0.61 (0.49)	**0.01**
ΔRrs5	0.67 (1.59)	0.39 (2.40)	0.23 (2.22)	0.58
ΔXrs5	-0.32 (1.13)	0.00 (1.19)	0.14 (0.57)	0.42

Rrs5 = Resistance at 5 Hz; Xrs = Reactance at 5 Hz; Fres = resonant frequency; dRrs/df = frequency dependency of resistance.

*between the three groups p, *adjusted for age*, *RSV etiology of bronchiolitis*, *current BMI z-score*, *current atopic eczema and use of inhaled corticosteroids during the preceding 12 months*. *p<0*.*05 marked in bold*

Similarly, girls carrying the A allele had significantly higher baseline Fres and lower dRrs/df compared to the non-carriers of allele A, and there was a trend towards lower Xrs at 5Hz but the association was not statistically significant. No signifcant differences were seen in hyper-reactivity parameters. (Table 7)

**Table 7:** Presence of *TLR7* rs179008 major allele A and baseline IOS results (z-scores) and exercise-induced changes (z-scores) in the 50 former bronchiolitis cases who were girls.

Parameter	A-allele (major) present	p-value*	A-allele not present
	n = 47		n = 3
	Mean (SD)		Mean (SD)
Rrs5	-0.08 (1.03)	0.90	-0.12 (1.10)
Xrs5	-0.68 (1.03)	0.08	0.49 (0.56)
Fres	2.28 (0.78)	**0.01**	0.89 (1.38)
dRrs/df	-1.03 (1.04)	**0.01**	0.61 (0.49)
ΔRrs5	0.57 (1.88)	0.61	0.57 (1.88)
ΔXrs5	-0.21 (1.15)	0.68	0.14 (0.57)

Rrs5 = Resistance at 5 Hz; Xrs = Reactance at 5 Hz; Fres = resonant frequency; dRrs/df = frequency dependency of resistance

*A-allele present vs. *A-allele not present*, *adjusted for age*, *RSV etiology of bronchiolitis*, *current BMI z-score*, *current atopic eczema and use of inhaled corticosteroids during the preceding 12 months*. *p<0*.*05 marked in bold*.

We also carried out an analysis that included the boys and girls in the same model. There were no significant associations with the IOS results when we compared 1) the 70 children in the male A and female A/A group with the 28 children in the male T and female TT group and 2) the 86 children in the male A, female A/A and female A/T group and the 12 children in the the male T and female TT group. (Data not shown)

In *TLR8* rs2407992, there were no significant associations between the presence of the G or C alleles and any baseline IOS parameters or hyper-reactivity parameters in boys or in girls (Data not shown).

The analyses were also carried out by including the boys and girls in the same model. There were no significant associations with the IOS results when we compared 1) the 46 children in the male G and female G/G group and the 52 children in the male C, female C/C and female G/C group and 2) the 67 children in the male G, female G/G and female G/C group and 31 children in the male C and female C/C group. (Data not shown)

In *TLR9* rs187084, there were no significant associations between genotypes or presence of alleles and IOS results in baseline lung function or bronchial hyper-reactivity measurements. (Data not shown)

## Discussion

This was an exploratory case-control study on lung function measured with IOS at a median age of 6.3 years in 103 children, who were prospectively followed-up since hospitalization for bronchiolitis at less than 6 months of age. The aim of the study was to explore if there are any associations between the TLRs encoding genes and lung function. There are two key findings. Firstly, *TLR4* and *TLR6* polymorphisms were associated with responses to exercise, but not with baseline lung function by IOS. Secondly, polymorphism in the X-chromosomally inherited *TLR7* was associated with abnormal baseline lung function by IOS in girls. This study reveals some novel but preliminary associations of TLR genetics and lung dysfunction in a high-risk post-bronchiolitis cohort.

TLRs are conserved pattern-recognizing proteins that function in the first line of innate immunity, either promoting or enhancing inflammatory processes and influencing the orientation of immunity into Th1 or Th2 directions. [30] In newborn infants, the Th1/Th2 balance is Th2-oriented and in atopy, the normal shift to the Th1-oriented balance does not happen, which leads to Th2 dominance and immunoglobulin E (IgE) production. [6] For instance, polymorphism in *TLR4* rs4986790 (Asp299Gly) was first seen to be associated with decreased airway reactivity to inhaled gram-negative bacterial lipopolysaccharide. [31] In later studies, exposures to environmental lipopolysaccharides in childhood were associated with less atopy, atopic asthma and rhinitis at school age. [10] Our finding that the *TLR4* rs4986790 wild type genotype was associated with some non-beneficial change in response to exercise suggests that polymorphism A to G may play a protective role controlling the emergence of hyper-reactivity.

A recent meta-analysis of TLR6 genetics provided inconsistent results about the association of *TLR6* polymorphisms with childhood asthma. [9] Polymorphism in *TLR6* rs5743810 was associated with atopic asthma in some studies, but most of the associations were non-significant or even protective from asthma. [9] Previous research in this cohort found an association between *TLR6* rs5743810 minor allele T and atopic eczema at 5 to 7 years of age. [22] Our present results suggest an association between *TLR6* rs5743810 C to T polymorphism and bronchial hyper-reactivity. Interestingly, the finding was robust to adjustment with atopic eczema, which suggests that the association between TLR6 and bronchial hyper-reactivity is not dependent on atopy.

TLR6 forms functional complexes with TLR1,TLR2, and TLR10, and therefore, we further analysed different combinations of the *TLR1*, *TLR2*, *TLR6* and *TLR10* genotypes. These supplementary analyses showed that the *TLR6* variant genotype was associated with bronchial hyper-reactivity jointly with the wild *TLR1*, *TLR2* and *TLR10* genotypes. In addition, the combination of the four wild genotypes *TLR1*, *TLR2*, *TLR6* and *TLR10* was associated with less airway reactivity. This suggests that wild *TLR2* subfamily genotypes may even be protective for later airway hyper-reactivity, and, in particular, *TLR6* rs5743810 variant genotypes may be associated with increased airway reactivity after infant bronchiolitis. The result is in line with our previous clinical observations in the same cohort. Only two children (8%) with wild genotypes in the *TLR1*, *TLR2* and *TLR6* genes had asthma during the first six years of life, compared to 30% in those with variant genotypes. [22]

The endoplasmic retinaculum TLRs 3, 7, 8 and 9 have not been as widely studied as other TLRs, although they play an important role in detecting respiratory viruses, such as single strand RNA structures in RSV and rhinoviruses. [5] There is some evidence that polymorphisms of *TLR7* and *TLR8* genes may have an association with asthma in adolescents and adults. [32] That association was not gender-specific, although some *TLR7* and *TLR8* genes, like *TLR7* rs179008 and *TLR8* rs2407992, which were applied in the present study, are located in the X chromosome. In the present lung function study, there were no significant associations between *TLR7* or *TLR8* polymorphisms and lung function or airway reactivity by IOS in boys, but some associations were found in girls. In *TLR7* rs179008, the variant A/T genotype was associated with highest baseline Fres and lowest dRs/df, reflecting mild peripheral obstruction or dynamic restriction. The same changes were seen with presence of the *TLR7* rs179008 major allele A. This finding might reflect the lability of the small airways at preschool age, and although there were no significant differences in the main parameters, the reactance at 5Hz was lowest in those having the allele A, which suggests decreased lung elastic properties. These are preliminary findings of gender-specific structural differences in the airways or gender-specific differences in the inflammatory processes regulated by the *TLR7* gene.

The main shortcoming of this study was the small number of cases for a genetic study. In addition, the cohort size was insufficient for further stratified analyses of defined risk groups. Therefore, there is a risk of type-two statistical errors. On the other hand, analyzing many different IOS parameters as continuous variables between different genotype-specific and allele-specific subgroups increased the risk of type-one statistical errors. The 12 (12.2%) children who were using ICSs at the time of the study were included in the analyses, which might explain our somewhat lower figures for post-exercise hyper-reactivity. On the other hand, the analyses were adjusted for the use of ICSs, and when we compared the results of adjusted and non-adjusted analyses, the conclusions were similar.

We explored the possible associations of polymorphisms of 9 different TLRs, including genotypes and haplotypes in separate analyses, and used 4 lung function outcomes and 2 airway reactivity outcomes. This means 72 individual tests for lung function and 36 for airway reactivity outcomes. We considered our study as an exploratory study to find preliminary evidence for associations, if present, which needs to be confirmed or rejected in coming confirmatory studies. Therefore, we did not regard any multiplicity adjustments necessary. [33,34]

The strengths of our study were the long, prospective, 5 to 7 years follow-up after hospitalization for viral bronchiolitis in infancy. Though the present design was a case-control study, the long prospective follow-up upgraded the reliability of the data on confounding and disease modifying factors. In addition, the material is unique since the follow-up started before six months of age. At preschool age, we were able to determine lung function by IOS in more than 95% of the participants. All study subjects were of Finnish origin, which is a benefit in genetic studies. The IOS results were compared to new, up-to-date, national reference values that were population-based, height-adjusted and age-specific. [16]

## Conclusions

In conclusion, the *TLR1*, *TLR2*, *TLR3*, *TLR4*, *TLR6*, *TLR8*, *TLR9* or *TLR10* polymorphisms that we studied did not show significant associations with lung function, as measured by IOS at five to seven years of age after infant bronchiolitis. However, we found in this exploratory study that *TLR4*, and *TLR6* in particular, might be associated with airway reactivity, and that in girls *TLR7* might be associated with decreased lung function. To confirm these preliminary results the corresponding hypotheses need to be tested in further confirmatory studies

## Supporting Information

S1 Dataset 98 children at age 5–7 years after bronchiolitis. (XLSX)
